# Detection and genome characterization of four novel bat hepadnaviruses and a hepevirus in China

**DOI:** 10.1186/s12985-017-0706-8

**Published:** 2017-02-22

**Authors:** Bo Wang, Xing-Lou Yang, Wen Li, Yan Zhu, Xing-Yi Ge, Li-Biao Zhang, Yun-Zhi Zhang, Claus-Thomas Bock, Zheng-Li Shi

**Affiliations:** 1 CAS Key Laboratory of Special Pathogens and Biosafety and Center for Emerging Infectious Diseases, Wuhan Institute of Virology, Chinese Academy of Sciences, Wuhan, China; 2 Guangdong Institute of Applied Biological Resource, Guangzhou, 510260 China; 3 Laboratory for Zoonosis Control and Prevention, Yunnan Institute of Endemic Diseases Control and Prevention, Dali, 671000 China; 4 Department of Infectious Diseases, Robert Koch Institute, Berlin, Germany

**Keywords:** Bat, Natural reservoir, Hepadnavirus, Hepevirus, Genome characterization

## Abstract

**Background:**

In recent years, novel hepadnaviruses, hepeviruses, hepatoviruses, and hepaciviruses have been discovered in various species of bat around the world, indicating that bats may act as natural reservoirs for these hepatitis viruses. In order to further assess the distribution of hepatitis viruses in bat populations in China, we tested the presence of these hepatitis viruses in our archived bat liver samples that originated from several bat species and various geographical regions in China.

**Methods:**

A total of 78 bat liver samples (involving two families, five genera, and 17 species of bat) were examined using nested or heminested reverse transcription PCR (RT-PCR) with degenerate primers. Full-length genomic sequences of two virus strains were sequenced followed by phylogenetic analyses.

**Results:**

Four samples were positive for hepadnavirus, only one was positive for hepevirus, and none of the samples were positive for hepatovirus or hepacivirus. The hepadnaviruses were discovered in the horseshoe bats, *Rhinolophus sinicus* and *Rhinolophus affinis*, and the hepevirus was found in the whiskered bat *Myotis davidii*. The full-length genomic sequences were determined for one of the two hepadnaviruses identified in *R. sinicus* (designated BtHBVRs3364) and the hepevirus (designated BtHEVMd2350). A sequence identity analysis indicated that BtHBVRs3364 had the highest degree of identity with a previously reported hepadnavirus from the roundleaf bat, *Hipposideros pomona*, from China, and BtHEVMd2350 had the highest degree of identity with a hepevirus found in the serotine bat, *Eptesicus serotinus*, from Germany, but it exhibited high levels of divergence at both the nucleotide and the amino acid levels.

**Conclusions:**

This is the first study to report that the Chinese horseshoe bat and the Chinese whiskered bat have been found to carry novel hepadnaviruses and a novel hepevirus, respectively. The discovery of BtHBVRs3364 further supports the significance of host switches evolution while opposing the co-evolutionary theory associated with hepadnaviruses. According to the latest criterion of the International Committee on Taxonomy of Viruses (ICTV), we hypothesize that BtHEVMd2350 represents an independent genotype within the species *Orthohepevirus D* of the family *Hepeviridae*.

## Background

Nearly 60% of emerging infectious diseases in humans are zoonotic, with up to 70% of them being found to originate from wildlife [1]. Bats have been identified as natural reservoirs of many viruses. Some of these viruses cause outbreaks of severe disease in humans [2], including the Ebola virus, the lyssavirus, the severe acute respiratory syndrome coronavirus, and henipaviruses [3]. Interestingly, these viruses rarely cause apparent clinical signs in bats [2]. Bats possess unique characteristics that may contribute to their ability to act as a major natural reservoir for viruses, including a high level of species diversity, a long lifespan, a high population density, and high levels of spatial mobility [4].

Previous studies mainly focused on bat-borne viruses that are transmitted via respiratory droplets [3]. However, in recent years, several hepatitis virus-related sequences, including those associated with hepadnaviruses, hepeviruses, hepatoviruses, and hepaciviruses, have been found in bats across the globe, indicating the importance of bats as the natural reservoirs of these viruses [5–9].

Hepatitis viruses include hepatitis viruses A, B, C, D, and E, which cause human hepatitis diseases. Hepatitis A virus (HAV) is classified as belonging to the genus *Hepatovirus* in the family *Picornaviridae*. Hepatitis B virus (HBV) is classified as belonging to the genus *Orthohepadnavirus* in the family *Hepadnaviridae*. Hepatitis C virus (HCV) is classified as belonging to the genus *Hepacivirus* in the family *Flaviriridae*. Hepatitis D virus (HDV) is considered to be a subviral satellite because it can only propagate in the presence of HBV. Hepatitis E virus (HEV) is classified as belonging to the genus *Orthohepevirus* in the family *Hepeviridae*. Hepatovirus-related sequences have been identified in 13 species of bat collected in North America, Europe, and Africa [5]. Hepadnavirus-related sequences have been discovered in five species of bat collected in Panama, Gabon, Myanmar, and China [6, 8–10]. Highly diverse hepacivirus-related sequences have been detected in 20 species of bat across the world [11]. Hepevirus-related sequences have been discovered in bats in Ghana, Panama, and Germany [7]. These results indicate that bats may be important reservoirs of these hepatitis viruses (Table 1).

**Table 1:** Hepatitis virus-like sequences detected in bats

Virus family	Order: Chiroptera			Sampling site (s) (year)	Isolation source	Genomic sequence	Reference
	Family	Genera	Species				
Hepatoviridae	Emballonuridae	*Coleura*	*C. afra*	Ghana (2011), Gabon (2009)	Feces, blood	Full	[5]
	Pteropodidae	*Eidolon*	*E. helvum*	Ghana (2010/2011)	Blood	Full	
	Natalidae	*Natalus*	*N. lanatus*	Costa Rica (2010)	Feces	Full	
	Rhinolophidae	*Rhinolophus*	*R. landeri*	Ghana (2011)	Feces	Full	
			*R. ferrumequinum*	Romania (2008/2009), Bulgaria (2008/2009), Luxemburg (2011)	Feces	Full	
		*Rhinolophus*	*R. hipposideros*	Bulgaria (2008/2009), Spain (2010)	Feces	Full	
	Vespertilionidae	*Glauconycteris*	*G. spec.*	Côte d’Ivoire (2013)	Intestines	Full	
		*Miniopterus*	*M. cf. manavi*	Madagascar (2014)	Liver	Full	
			*M. schreibersii*	Romania (2008)	Feces	Full	
		*Myotis*	*M. dasycneme*	Germany (2008)	Feces	Full	
			*M. myotis*	Romania (2008), Germany (2008/2010)	Feces	Full	
		*Nyctalus*	*N. noctula*	Romania (2008/2009), Germany (2009)	Feces	Full	
		*Pipistrellus*	*P. kuhlii*	Ukraine (2010/2011)	Feces	Full	
Hepadnaviridae	Vespertilionidae	*Miniopterus*	*Miniopterus spp.*	Myanmar (2010)	Liver	Full	[8]
	Hipposideridae	*Hipposideros*	*H. pomona*	China (2011)	Liver	Full	[9]
			*H. cf. ruber*	Gabon (2009)	Blood	Full	[6]
	Phyllostomidae	*Uroderma*	*U. bilobatum*	Panama (2010/2011)	Blood	Full	
	Rhinolophidae	*Rhinolophus*	*R. alcyone*	Gabon (2009)	Blood	Full	
			*R. sinicus*	*China (2012)*	*Liver*	*Full*	*This study*
			*R. affinis*	*China (2013)*	*Liver*	*Partial*	
Hepaciviridae	Emballonuridae	*Taphozous*	Not identified	Cameroon (2010)	Blood	Partial	[11]
	Hipposideridae	*Hipposideros*	*H. vittatus*	Kenya (2010)	Blood	Full	
			*H. gigas*	Nigeria (2008/2010)	Blood	Partial	
	Molossidae	*Chaerephon*	Not identified	Cameroon (2010), Kenya (2010)	Blood	Partial	
		*Mops*	*M. condylurus*	DRC (2011)	Blood	Full	
		*Otomops*	*O. martiensseni*	Kenya (2010)	Blood	Full	
		*Nyctinomops*	*N. macrotis*	Mexico (2011)	Blood	Partial	
	Phyllostomidae	*Artibeus*	*A. watsoni*	Mexico (2011)	Blood	Partial	
		*Carollia*	*C. perspicillata*	Guatemala (2010), Mexico (2011)	Blood	Full	
		*Desmodus*	*D. rotundus*	Guatemala (2010),	Blood	Partial	
		*Glossophaga*	*G. commissarisi*	Mexico (2011)	Blood	Partial	
		*Sturnira*	*S. lilium*	Guatemala (2010)	Blood	Full	
			*S. ludovici*	Mexico (2011)	Blood	Partial	
		*Trachops*	*T. cirrhosus*	Mexico (2011)	Blood	Partial	
	Pteropodidae	*Eidolon*	*E. helvum*	Cameroon (2010), DRC (2011)	Blood	Full	
		*Epomophorus*	*E. labiatus*	Kenya (2010)	Blood	Partial	
		*Megaloglossus*	*M. woermanni*	DRC (2011)	Blood	Full	
		*Rousettus*	*R. aegyptiacus*	Kenya (2010), Nigeria (2008/2010)	Blood	Full	
	Vespertilionidae	*Scotoecus*	Not identified	Kenya (2010)	Blood	Partial	
		*Scotophilus*	*S. dingani*	Kenya (2010)	Blood	Full	
			*S. nigrita*	Nigeria (2008/2010)	Blood	Partial	
	Unknown			Nigeria (2008/2010), Mexico (2010/2011)	Blood	Partial	
Hepeviridae	Hipposideridae	Hipposideros	*H. abae*	Ghana (2009)	Feces	Partial	[7]
	Phyllostomidae	Vampyrodes	*V. caraccioli*	Panama (2011)	Blood	Partial	
	Vespertilionidae	Eptesicus	*E. serotinus*	Germany (2008)	Liver	Full	
		Myotis Myotis	*M. bechsteinii*	Germany (2008)	Feces	Partial	
			*M. daubentonii*	Germany (2008)	Feces	Partial	
			*M. davidii*	*China (2011)*	*Liver*	*Full*	*This study*

*DRC* Democratic Republic of the Congo

There are around 120 species of bat in China; however, only limited information has been reported regarding the hepatitis viruses, a novel Orthohepadnavirus in pomona roundleaf bats from Yunnan province was identified in 2015 [9]. In this study, we report the discovery of four novel hepadnaviruses and a hepevirus in our archived bat liver samples that had been collected from several bat species and various geographical regions in China.

## Methods

### Samples

A total of 78 liver tissue samples were collected from dead bats caused by accident during sampling, which comprised two families, five genera, and 17 species, and used for virus screening (Table 2). Different tissues (heart, liver, spleen, lung, kidney, brain and intestine) were collected separately and used for analyzing virus tissue tropism. The animals were firstly identified based on their morphology and then the species that they belonged to were further confirmed using DNA sequencing of the mitochondrial cytochrome b (*CytB*) gene following previously described methods [12].

**Table 2:** Detection of hepadnavirus and hepevirus in bats in China between 2008 and 2013

Family	Genus	Species	No. of samples	No. of hepadnavirus positive samples	No. of hepevirus positive samples	Sampling site (s) (year)
*Vespertilionidae*	*Myotis*	*M. adversus*	4			Yunnan (2008)
		*M. pilosus*	7			Yunnan (2008)
		*M. chinensis*	1			Hubei (2008)
		*M. davidii*	12		1	*Hubei (2011)*, Yunnan (2011)
		*M. ikonnikovi*	3			Yunan (2009), Hubei (2011)
		*M. formosus*	1			Yunnan (2011)
	*Miniopterus*	*M. fuliginosus*	1			Henan (2010)
	*Aselliscus*	*A. stoliczkanus*	1			Yunnan (2012)
*Rhinolophidae*	*Rhinolophus*	*R. sinicus*	19	2		Hubei (2008/2011), Sichuan (2011), *Yunnan (2009/2012/2013)*
		*R. monoceros*	3			Hubei (2011), Chongqing (2011)
		*R. affinis*	7	2		Henan (2010), Hubei (2011) *Yunnan (2012/2013)*,
		*R. pusillus*	4			Yunnan (2012/2013)
		*R. pearsonii*	1			Chongqing (2011)
	*Hipposideros*	*H. pratti*	2			Hubei (2010)
		*H. armiger*	4			Sichuan (2011)
		*H. pomona*	7			Yunnan (2013)
		*H. larvatus*	1			Yunnan (2011)
Total	5 genera	17 species	78	4	1	

### RNA extraction and PCR

RNA was extracted from tissue using the QIAamp Viral RNA Mini Kit (Qiagen, Hilden, Germany) following manufacturer’s instructions, and cDNA was synthesized using Moloney Murine Leukemia Virus (M-MLV) Reverse Transcriptase (Promega, Madison, WI, USA). The extracted RNA from liver was tested by nested or heminested reverse transcription PCR (RT-PCR) using degenerate primers based on the conserved domain of the RNA-dependent RNA polymerase (RdRp) gene of viruses in the genus *Hepatovirus*, the polymerase gene of viruses in the family *Hepadnaviridae*, the RdRp gene of viruses in the genus *Hepacivirus* [11], and the RdRp gene of viruses in the family *Hepeviridae* [7] (Table 3). Standard precautions were taken to avoid contamination of the PCR procedure, and no false-positives were observed in the negative controls. The PCR products underwent gel purification with MinElute Gel Extraction Kit (Qiagen, Germany) and they were sequenced with both forward and reverse primers using the 3100 Sequencer (ABI, Waltham, MA, USA).

**Table 3:** Primers used for virus RT-PCR screening and virus quantification

Primer	Sequence (5′-3′)^a^	Polarity	Targeted virus	Reference
HAV-3D-F1	CYTATHTRAARGATGAGCTKAGA	+	Hepatovirus	This study
HAV-3D-F2	ACRTCATCICCRTARCAIAGRA	+		
HAV-3D-R1	RTCIAARACWAGRGCNATYG	-		
HAV-3D-R2	TACCWAATCATRAATGGACT	-		
HBV-pol-F1	TAGACTSGTGGTGGACTTCTC	+	Hepadnavirus	This study
HBV-pol-F2	AGTRAAYTGAGCCAGGAGAAAC	+		
HBV-pol-R1	TGCCATCTTCTTGTTGGTTC	-		
HBV-pol-R1	CATATAASTRAAAGCCAYACAG	-		
BHV-1-F1	GTAGCGGAGAAGATGTATCTGGG	+	Hepacivirus	[11]
BHV-1-R1	GCCTTAGCCTTGAGAAAGCAGGTGAT	+		
BHV-1-F2	GAGAAGATGTATCTGGGGGACGT	+		
BHV-1-R2	AGAAAGCAGGTGATGGTATTGCC	+		
BHV-2-F1	CCAAARGTWGTBAAGGCTGTGCT	-		
BHV-2-R1	ACTTTGAKCCASGCAGTKARACAGTT	-		
BHV-2-F2	GCTGTGCTSAAGGAMGAGTACGGCT	+		
BHV-2-R2	CCASGCAGTKARACAGTTACTRGAG	-		
DE-F4228	ACYTTYTGTGCYYTITTTGGTCCITGGTT	+	Hepevirus	[7]
DE-R4598	GCCATGTTCCAGAYGGTGTTCCA	-		
DE-R4565	CCGGGTTCRCCIGAGTGTTTCTTCCA	-		
BtHEV-qF	ATGTCCGTGTTCAGGTTCC	+	Bat hepevirus	This study
BtHEV-qR	GCCAACCCTCATTTGCAAC	-		
BtHBV-qF	TGTTGGTTCTCCTGGATTGGAG	+	Bat hepadnaviruses	This study
BtHBV-qR	TGAAGGAATGGGCCAGCAGGTG	-		

R: G/A; Y: C/T; S: G/C; W: A/T; M: A/C; K: G/T; H: A/C/T; N: A/T/C/G; I: inosine

### Genomic sequencing

The complete genomic sequences of one hepadnavirus strain and one hepevirus strain were amplified using PCR with degenerate primers (the primers are available upon request). The genome ends were amplified using a 5′-Full RACE Kit (TaKaRa, Japan). The PCR products underwent gel purification with MinElute Gel Extraction Kit (Qiagen, Germany) and they were sequenced with both forward and reverse primers using the 3100 Sequencer. The sequencing chromatograms were inspected for overlapping multicolor peaks, which are an indicator of sequence heterogeneity in the amplicons. The PCR products were cloned using the pGEM-T Easy Vector System (Promega, Germany) and at least three clones for each PCR fragment were sequenced to obtain a consensus sequence.

### Sequence analysis

**Fig. 1:**
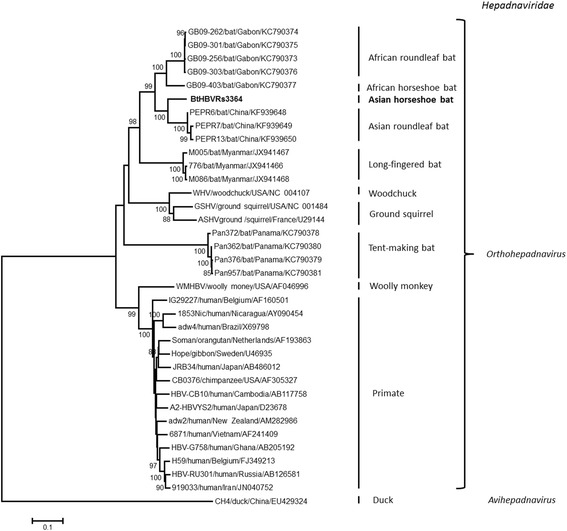
Phylogenetic analysis of bat hepadnavirus based on the full-length genomic sequences. Maximum likelihood phylogenetic tree was constructed based on the complete genomes of BtHBVRs3364 (in bold) and representative members of the family *Hepadnaviridae* using the Hasegawa-Kishino-Yano substitution model and complete deletion option in MEGA version 7. The values at the nodes indicate the bootstrap values (using 1,000 replications). The branches are labeled with the strain designation, the host species, and the GenBank accession number. The classification of the family *Hepadnaviridae* is indicated on the right

**Fig. 2:**
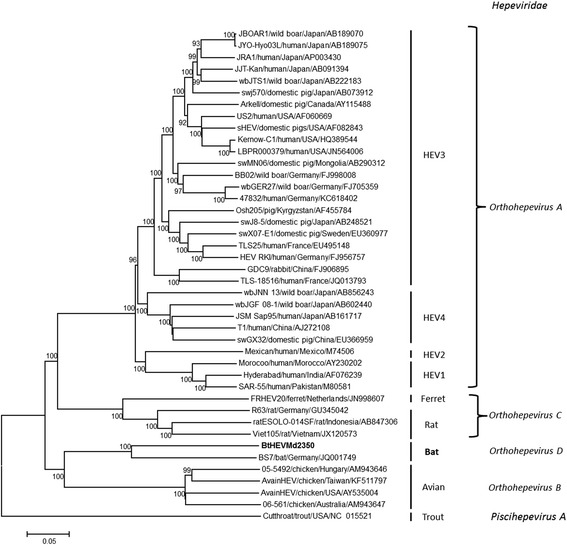
Phylogenetic analysis of bat hepevirus based on the full-length genomic sequences. Neighbor joining phylogenetic tree was constructed based on the alignment of the complete genomes of BtHEVMd2350 (in bold) and representative members of the family *Hepeviridae* using nucleotide percentage distance substitution matrix and complete deletion option implemented in MEGA version 7. The values at nodes indicate the bootstrap values (using 1,000 replications). The branches are labeled with the strain designation, the host species, and the GenBank accession number. The classification of the family *Hepeviridae* is indicated on the right

The preliminary sequence management and analysis were carried out using Geneious version 9.1.3 (Biomatters Ltd., Auckland, New Zealand) and the sequence alignment and editing were performed using MAFFT [13]. The phylogenetic analysis of hepadnavirus used the neighbor-joining (NJ) method with Hasegawa-Kishino-Yano substitution model and complete deletion option and hepevirus used the maximum-likelihood (ML) method with the nucleotide percentage distance substitution matrix and the complete deletion option in MEGA version 7 [14]. The sequences and GenBank accession numbers of the representative viruses in the families *Hepadnaviridae* and *Hepeviridae* used in the phylogenetic analyses are presented in Figs. 1 and 2.

### Quantification real-time PCR

Virus load of bat hepevirus and hepadnaviruese of different tissues was measured by using photometrically quantified in vitro RNA transcripts and specific real-time RT-PCR primers (Table 3). Quantification was done by using 5 μL of RNA extract, 300 nM each primer, using the One Step SYBR PrimeScript™ PLUS RT-PCR Kit (TaKaRa, Japan). Cycling in a Biorad CFX Connect instrument involved the following steps: 42 °C for 5 min, 95 °C for 10 s, and 40 cycles of 95 °C 5 s and 60 °C 20 s with measurement of fluorescence.

## Results

### Detection of four hepadnaviruses and a hepevirus in bat liver samples

**Fig. 3:**
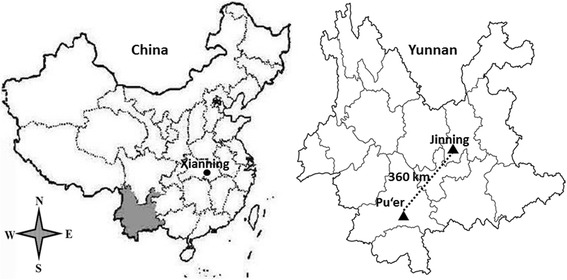
Representation map of China and Yunnan province. *Circle* indicates the sampling site where the hepevirus (BtHEVMd2350) was detected. *Triangles* indicates the sampling sites where the hepadnaviruses were detected

Among the 78 bat liver samples, four were positive for hepadnavirus from Jinning city, Yunnan province and only one was positive for hepevirus from Xianning city, Hubei province (Fig. 3). However, none were positive for hepatovirus or hepacivirus. The nucleotide sequences of the four novel hepadnaviruses and the hepevirus described in this study are available from GenBank under the accession numbers KX513949–KX513953.

### Sequence analysis of the bat hepadnavirus

All four of the hepadnavirus-positive samples were from horseshoe bats, two each from *R. sinicus* (designated BtHBVRs3364 and BtHBVRs3366) and *R. affinis* (designated BtHBVRa4325 and BtHBVRa4328) (Table 2). The four partial polymerase gene sequences had 92.1–97.5% nucleotide sequence identity and they were found to be closely related to the roundleaf bat hepadnavirus from Yunnan province, China, with nucleotide identities of 88.8–95.5% [9].

The full-length genomic sequence of a sample from *R. sinicus* (designated bat HBV Rs3364, or BtHBVRs3364) was determined and it was found to have a length of 3,272 nucleotides. The virus has an identical genomic organization to other hepadnaviruses, with four open reading frames (ORFs) encoding the surface (S), polymerase (P), core (C), and X proteins. In addition, the typical direct repeat (DR) sequences for viral genome replication and the secondary structure Ɛ–loops for viral reverse transcription were present in the BtHBVRs3364 genome. A detailed comparison of the full-length genomic sequence and the ORFs of the virus with other known hepadnaviruses is shown in Table 4. The results showed that the four genes of BtHBVRs3364 have the highest degree of identity with the roundleaf bat hepadnavirus from Yunnan province, at both the nucleotide and the amino acid levels [9]. Notably, we found large differences between BtHBVRs3364 and other hepadnaviruses from the African horseshoe and roundleaf bats, the long-fingered bat from Myanmar, and the tent-making bat from Panama.

**Table 4:** Nucleotide and amino acid sequence identity between BtHBVRs3364 and representative orthohepadnavirus strains^a^

Hepadnavirus (no. of strains compared)	Degree of identity (%)								
	Full genome	S gene (1–672)		X gene (1217–1645)		C gene (1657–2301)		P gene (2147–1475)	
	Nucleotides	Nucleotides	Amino acids	Nucleotides	Amino acids	Nucleotides	Amino acids	Nucleotides	Amino acids
Asian roundleaf bat hepadnavirus (3)	81.2–81.9	90.5–93.9	83.5–90.2	86.7	75.4	85.2–85.3	91.2–91.7	80.1–80.9	77.4–77.9
African horseshoe bat hepadnavirus (1)	72.2	89.0	84.8	78.8	65.5	75.2	77.9	71.8	65.9
African roundleaf bat hepadnavirus (4)	72.0–72.1	89.0–89.2	86.2	81.8–82.1	70.4–71.1	76.9–77.1	77.9	72.3–72.4	65.6–66.0
Long-fingered bat hepadnavirus (3)	69.0–69.2	79.7–80.1	67.4	69.2–70.2	49.3–51.4	74.2–74.5	75.6–76.0	68.1–68.3	61.5–61.8
Tent-making bat hepadnavirus (4)	56.7–56.8	73.5–74.2	61.0–61.9	58.9–59.3	43.4–44.1	51.6	49.5	56.8–57.1	49.1–49.2
Primate HBV (15)	59.5–61.1	76.5–77.8	65.0–66.8	62.1–64.9	46.5–52.1	61.0–67.9	63.0–66.5	57.0–59.7	50.2–52.0
Woolly monkey hepadnavirus (1)	61.5	75.3	67.1	63.2	50.0	64.2	61.4	60.3	51.9
Ground squirrel hepadnavirus (2)	64.0–65.1	78.7–78.8	67.9–68.3	60.6–63.9	45.1–46.5	71.1–71.4	70.9–74.0	58.9–59.1	49.3–50.2
Woodchuck hepadnavirus (1)	59.8	77.9	67.4	63.9	47.2	69.8	72.5	58.8	50.9
Duck hepadnavirus (1)	31.1	44.1	28.7	NA	NA	31.3	20.9	35.1	23.2

^a^The sequences were aligned using MAFFT. The evolutionary analyses were conducted using MEGA version 7. The GenBank accession numbers are as follows: KF939648, KF939649, and KF939650 for the Asian roundleaf bat hepadnaviruses; KC790377 for the African horseshoe bat hepadnavirus; KC790374, KC790375, KC790373, and KC790376 for the African roundleaf bat hepadnaviruses; KC790378, KC790379, KC790380, and KC790381 for the Tent-making bat hepadnaviruses; AF160501, AY090454, X69798, AF193863, U46935, AB486012, AF305327, AB117758, D23678, AM282986, AF241409, AB205192, FJ349213, AB126581, and JN040752 for the Primate HBVs; AF046996 for the woolly monkey hepadnavirus; NC 001484 and U29144 for the ground squirrel hepadnaviruses; NC_004107 for the woodchuck hepadnavirus; and EU429324 for the duck hepadnaviruses. *NA* not available

### Phylogenetic analysis of the bat hepadnavirus

A phylogenetic tree was constructed based on the alignment of the full-length genomic sequence of BtHBVRs3364 with those of representative hepadnavirus strains available in GenBank. As shown in Fig. 1, the previously reported bat hepadnaviruses formed three clusters, with clear specificities for particular hosts. Although BtHBVRs3364 clustered with the bat hepadnaviruses, it formed an independent branch. Interestingly, the BtHBVRs3364 detected in the horseshoe bat is phylogenetically closer to viruses from the Asian roundleaf bat compared to viruses from the African horseshoe bat, despite the fact that it was found in an Asian horseshoe bat.

### Sequence analysis of the bat hepevirus

One sample found in the whiskered bat, *M. davidii*, from Hubei province was positive for hepevirus (designated bat HEV Md2350, or BtHEVMd2350). The genomic sequence of BtHEVMd2350 was found to have a length of 6,607 nucleotides (excluding the poly(A) tail at the 3′ end). This is slightly shorter than BS7 (which has a genomic sequence length of 6,671 nucleotides), the only reported bat hepevirus with a fully sequenced genome, which was identified from the serotine bat, *Eptesicus serotinus*, in Germany [7]. BtHEVMd2350 was found to have a 5′ untranslated region (UTR) of 33 nucleotides and a 3′ UTR of 76 nucleotides. The three unique ORFs that are found in other members of the family *Hepeviridae* were also found in BtHEVMd2350: ORF1 encodes a nonstructural polyprotein that includes the RdRp, ORF2 encodes the capsid protein, and ORF3 encodes a multifunctional protein. Notably, most of the elements and domains characterized in BS7 could be found in BtHEVMd2350, but with a high level of divergence (Table 5).

**Table 5:** Nucleotide and amino acid sequence identity between BtHEVMd2350 and representative hepevirus strains^a^

Hepevirus (no. of strains compared)	Degree of identity (%)						
	Full genome	ORF1 (genome positions 56–4699)		ORF2 (genome positions 4700–6607)		ORF3 (genome positions 4779–5192)	
	Nucleotides	Nucleotides	Amino acids	Nucleotides	Amino acids	Nucleotides	Amino acids
Bat hepevirus (1)	67.8	65.5	72.0	70.9	79.2	73.2	44.3
Avian hepevirus (4)	50.9–51.7	48.2–49.0	44.3–44.5	46.0–47.3	44.4–44.9	19.9–21.1	5.3–6.1
HEV genotype 3 (22)	44.6–46.0	43.4–45.5	39.1–40.9	47.4–49.9	44.6–47.4	30.7–31.8	14.8–18.9
HEV genotype 4 (5)	45.2–46.0	44.9–45.9	40.4–41.0	47.5–48.2	45.8–47.1	30.0–31.6	14.6–16.3
HEV genotype 1 (3)	45.6–45.9	45.0–45.5	40.5–41.1	48.7–49.3	46.6–46.9	31.9–32.6	17.1
HEV genotype 2 (1)	45.6	45.3	40.8	49.7	47.1	31.6	17.1
Rodent hepevirus (3)	44.0–44.4	45.9–46.1	41.6–42.0	47.9–48.4	45.4–46.7	25.2–27.4	9.0
Ferret hepevirus (1)	44.0	46.4	42.6	48.3	46.0	25.5	8.0–12.8
Trout hepevirus (1)	33.7	34.9	24.5	30.2	15.5	21.1	9.8

^a^The sequences were aligned using MAFFT. The evolutionary analyses were conducted using MEGA version 7. The GenBank accession numbers are as follows: JQ001749 for the bat hepevirus; AM943646, AM943647, KF511797, and AY535004 for the avian hepeviruses; AB189070, AB189075, AP003430, AB091394, AB222183, AB073912, AY115488, AF060669, AF082843, HQ389544, JN564006, AB290312, FJ998008, FJ705359, KC618402, AF455784, AB248521, EU360977, EU495148, FJ956757, FJ906895, and JQ013793 for the HEVs genotype 3; AB856243, AB602440, AB161717, AJ272108, and EU366959 for the HEVs genotype 4; AY230202, AF076239, and M80581 for the HEVs genotype 1; M74506 for HEV genotype 2; GU345042, AB847306, and JX120573 for the rodent hepeviruses; JN998607 for the ferret hepevirus; and NC_015521 for the trout hepevirus

### Phylogenetic analysis of the bat hepevirus

A phylogenetic tree was constructed based on the alignment of the full-length genomic sequence of BtHEVMd2350 with those of representative full-length hepevirus genomic sequences (Fig. 2). The results showed that bat hepeviruses (BtHEVMd2350 and BS7) cluster into a separate monophyletic clade within the family *Hepeviridae*.

### Quantification of novel viruses

**Fig. 4:**
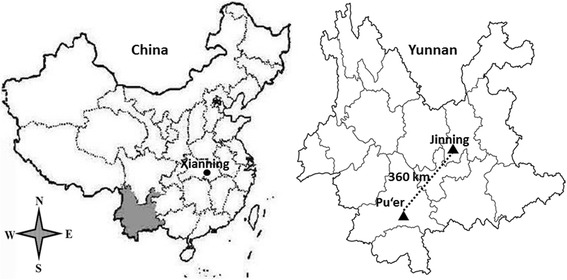
Tissue distribution of bat hepevirus and hepadnaviruses. Virus concentrations assessed by virus-specific one-step real-time RT-PCR using quantified in vitro-transcribed RNA controls

Viral load detected by qPCR in different tissues were presented in the Fig. 4. The highest viral load of the BtHEVMd2350 was found in the liver (1.9 × 10^10^ RNA copies per gram of tissue) and followed by spleen (7.3 × 10^8^ RNA copies per gram of tissue), intestine and kidney, but not detectable in the brain. For bat hepadnavirus, the highest viral load was found in the liver of BtHBVRs3364 (2.0 × 10^10^ RNA copies per gram of tissue), the virus load of tissues of BtHBVRs3366, BtHBVRa4325, and BtHBVRa4328 were relatively similar (medien, 6.2 × 10^6^ RNA copies per gram of tissue; range, 4.9 × 10^5^ to 2.7 × 10^10^ RNA copies per gram of tissue).

## Conclusions and discussion

Since the discovery of genetically diverse hepatitis virus-related sequences in bats, bats have been considered to be important natural reservoirs for hepatitis viruses, and potential sources of human diseases [10]. However, these hypotheses need to be proved by screening more bat samples from across the globe for hepatitis viruses. In this study, we screened for hepatitis viruses in bats from China and discovered four novel hepadnaviruses circulating in two species of horseshoe bat in Jinning city, Yunnan province and one hepevirus in the whiskered bat *M. davidii* in Xianning city, Hubei province. The full-length genomic sequences of one of the two hepadnaviruses from *R. sinicus* and the hepevirus from *M. davidii* were determined.

The phylogenetic analysis indicates that the bat hepadnavirus found in this study is closely related to roundleaf bat hepadnaviruses, which were discovered in Pu’er city, Yunnan province in 2011 [9], but shows remarkable divergence when compared to the African horseshoe bat, despite the fact that it was found in an Asian horseshoe bat. A similar phylogenetic relationship was found between hepadnaviruses from the African roundleaf bat and the African horseshoe bat [6], indicating the separate evolution of these viruses and their hosts.

Regarding the bat hepevirus, the phylogenetic analysis indicates that the known bat hepeviruses are highly divergent from other mammalian hepeviruses and that they form an independent branch in the family *Hepeviridae*. According to the latest proposal of the ICTV in 2016, amino acid distances of concatenated ORF1 and ORF2 (lacking hypervariable regions) greater than 0.088 could then act as threshold to demarcate intra- and inter- genotype distances [15]. The hepevirus detected in the whiskered bat, *M. davidii*, and that found in the German serotine bat, *E. serotinus* (the only reported bat hepevirus with a full-length genome) shared significant diversity from both nucleotide and amino acid levels, we propose that they can be grouped into the species *Orthohepevirus D* which is divided into two genotypes: D1 and D2.

Our results provide further evidence to support the theory regarding the long-term co-evolution of hepadnaviruses and hepeviruses with their hosts, and the theory that bats act as major natural reservoirs for these hepatitis viruses. Our results have limitations due to the small sample size used, which was a result of the protection of bat populations in China, as bats play important roles in the pollination of plants and in pest control, as they feed on insects. However, based on our discovery of hepatitis viruses in bats, it is expected that there are many more hepatitis viruses circulating in numerous bat species and in various geographic regions. In order to obtain larger sample sizes, non-invasive methods of virus detection should be considered for future studies.

## Abbreviations

C: Core
HAV: Hepatitis A virus
HBV: Hepatitis B virus
HCV: Hepatitis C virus
HDV: Hepatitis D virus
HEV: Hepatitis E virus
ORFs: Open reading frames
P: Polymerase
RT-PCR: Reverse transcription polymerase chain reaction
S: Surface
